# Effect of Hybrid Talc-Basalt Fillers in the Shell Layer on Thermal and Mechanical Performance of Co-Extruded Wood Plastic Composites

**DOI:** 10.3390/ma8125473

**Published:** 2015-12-08

**Authors:** Runzhou Huang, Changtong Mei, Xinwu Xu, Timo Kärki, Sunyoung Lee, Qinglin Wu

**Affiliations:** 1 College of Material Science and Engineering, Nanjing Forestry University, Nanjing 210037, China; runzhouhuang@gmail.com (R.H.); mei@njfu.edu.cn (C.M.); xucarpenter@aliyun.com (X.X.); 2 School of Renewable Natural Resources, Louisiana State University Agricultural Center, Baton Rouge, LA 70803, USA; 3 School of Energy Systems, Lappeenranta University of Technology, FI-53851 Lappeenranta, Finland; timo.karki@lut.fi; 4 Department of Forest Products, Korea Forest Research Institute, Seoul 130-712, Korea; nararawood@forest.go.kr

**Keywords:** composite, wood, thermoplastics, properties, analytical modeling, co-extrusion

## Abstract

Hybrid basalt fiber (BF) and Talc filled high density polyethylene (HDPE) and co-extruded wood-plastic composites (WPCs) with different BF/Talc/HDPE composition levels in the shell were prepared and their mechanical, morphological and thermal properties were characterized. Incorporating BFs into the HDPE-Talc composite substantially enhanced the thermal expansion property, flexural, tensile and dynamic modulus without causing a significant decrease in the tensile and impact strength of the composites. Strain energy estimation suggested positive and better interfacial interactions of HDPE with BFs than that with talc. The co-extruded structure design improved the mechanical properties of WPC due to the protective shell layer. The composite flexural and impact strength properties increased, and the thermal expansion decreased as BF content increased in the hybrid BF/Talc filled shells. The cone calorimetry data demonstrated that flame resistance of co-extruded WPCs was improved with the use of combined fillers in the shell layer, especially with increased loading of BFs. The combined shell filler system with BFs and Talc could offer a balance between cost and performance for co-extruded WPCs.

## 1. Introduction

Wood-plastic composites (WPC) are widely used in structural building applications including sheathings, decking, roof tiles, and window trims [1,2]. WPCs have improved thermal, mechanical and long-term creep performance compared with unfilled plastics, and enhanced durability compared with wood composites. However, it is still necessary to further improve their raw material input, processing, physical and mechanical properties. Among the techniques, using optimized filler systems and co-extrusion processing are considered to be cost-performance effective to improve overall properties of WPCs [1].

Various fillers with particulate (e.g., Talc, calcium carbonate) and fiber (e.g., glass, carbon, basalt fiber) geometries are available as a reinforcing phase for polymer composites [1]. Among the fillers, Talc, Mg_3_Si_4_O_10_(OH)_2_ with an average density of 2.75 g/cm^3^, is one of the most commonly used fillers in plastic-based composites [3,4]. The use of talc in composites can have a positive influence on modulus and strength, but may decrease toughness and ductility of filled plastics [5]. Talc is also widely used in wood and natural fiber plastic composites to reduce material cost and to improve stiffness and durability [1,6]. For example, it is shown that talc (up to 30 wt %) positively affected the modulus, strength, processing efficiency, creep and elastic recovery performance of WPCs [6]. More recently, Huang *et al.* [7,8] showed the effect of unmodified Talc particles on the mechanical and thermal expansion performance of Talc-filled high density polyethylene (HDPE) and co-extruded WPC with Talc-filled shells. The use of Talc in HDPE helped enhance its tensile, bending and dynamic modulus, but lowered its tensile and impact strength. Talc-filled HDPE, especially for composites with the Talc loading levels above the 30 wt % level, had reduced linear coefficient of thermal expansion (LCTE) values compared with that from the neat HDPE value. Coextruded WPCs with a relatively thick, less-stiff HDPE shell had decreased overall composite modulus and increased LCTE values [8]. However, the composite modulus and strength increased and LCTE values decreased as the talc loading levels in the shell increased.

Basalt fibers (BFs) are a relatively new and inexpensive fiber prepared by drawing a melted natural ore at a high temperature [9,10]. BFs have excellent corrosion and high temperature resistance, minimal moisture absorption, good sound absorption, and thermal insulation properties [11]. BFs are also a cost-effective and high-strength material for use in composites [12,13,14]. Chen *et al.* [15] investigated the effect of maleic anhydride grafted high-density polyethylene (MAPE) for enhancing mechanical properties of BF-WPCs. It was shown that the maximum values of the specific tensile and flexural strengths were achieved at a MAPE content of 5% to 8%. Recently, Wu *et al.* [16] successfully prepared BF filled HDPE and co-extruded WPCs with BF/HDPE composite shell and characterized their mechanical, morphological and thermal properties. The BFs with an average diameter of 7 μm and an organic surfactant surface coating were used in the study. Incorporating BFs into HDPE matrix substantially enhanced composite flexural, tensile and dynamic modulus. Compared to neat HDPE, BF/HDPE composites had reduced LCTE values. The use of the pure HDPE and BF/HDPE layers over a WPC core greatly improved composite impact strength. Both flexural and thermal expansion properties were enhanced with BF reinforced HDPE shells. 

Although individual classes of fillers or fibers can contribute some desirable properties as reinforcement fillers, hybrid fillers have attracted much attention as reinforced agents in composites with the intention of optimizing contributions from different types of fillers [17,18]. Hybrid filler reinforced composites form a complex system. The phenomena behind the property changes due to the addition of particulate fillers to the fiber reinforced thermoplastic composites is still less understood, especially for co-extruded WPC [19]. The objective of the study described in this paper was to investigate the effect of two individual fillers (*i.e*., BF *vs*. talc particulate) and their combinations on the morphological, mechanical and thermal expansion properties of the filled composites as potential shell material for coextruded WPC. The result of this study can help provide a fundamental base for developing new functional applications of co-extruded WPC with hybrid filler reinforced shells.

## 2. Experimental

### 2.1. Materials

BFs (density = 2.6 g/m^3^) were obtained from CETCO Oilfield Services (Broussard, LA, USA). The mean diameter of the fibers was approximately 7 μm with a standard deviation of 2.3 μm (Wu *et al.* 2014). The nominal length of the fibers was 2.5 cm, but the length varied in a random manner with some short fibers in the mix prior to compounding. Talc (oil absorption rate = 30 g/100 g Talc, density = 2.8 g/cm^3^, and particle size = 325 mesh) was supplied from the Fiber Glast Development Corp. (Brookville, OH, USA). HDPE (AD60-007, density = 0.963 g/cm^3^, melt index = 0.73 g/10 min at 190 °C/2.16 kg and softening temperature = 127 °C) was provided by ExxonMobil Chemical Co. (Houston, TX, USA). Pine wood flour (WF, 40-mesh particle size) was supplied by the American Wood Fibers Inc. (Schofield, WI, USA). A lubricant (TPW 306 from Struktol Co., Stow, OH, USA), and a maleated polyethylene (PE-g-MA) coupling agent (G2608, melt index = 8 g/10 min at 190 °C/2.16 kg, and acid number = 8 mg KOH/g from Eastman Chemical Co., Kingsport, TN, USA) were used as composite processing aids. 

### 2.2. Sample Preparation

#### 2.2.1. BF/Talc/HDPE Composites

Hybrid composites from BF, Talc and HDPE (BF/Talc/HDPE composites) were manufactured through two-step melt compounding using a Leistritz Micro-27 co-rotating parallel twin-screw extruder (Leistritz Corporation, Allendale, NJ, USA). In the first step, the BF/HDPE and Talc/HDPE mater-batch pellets were made separately with the fiber (BF) or particulate (Talc) to matrix weight ratio of 40/60. During the second extrusion, the BF/HDPE pellets were melt blended with Talc/HDPE pellets using the same extruder. Experimental design included a fixed filler to matrix weight ratio of 40:60 and variable ratios of BF/Talc namely 0/40, 4/36, 8/32, 12/28, 20/20 and 40/0 wt %.

Standard test samples for mechanical properties were made through injection molding, using a Plus 35 injection system from the Batenfeld of American Inc. (South Elgn, IL, USA). The blends were injection molded at injection temperatures of 180 to 185 °C and mold temperatures of 73 to 80 °C. Virgin HDPE control samples were directly molded at the injection temperature of 180 °C and the mold temperature of 73 °C. 

#### 2.2.2. Co-Extruded WPCs with BF and Talc Reinforced HDPE Shells

The composites were made with one core type and different shells containing various BF and talc contents in the HDPE matrix. The core formulation was Wood:HDPE:Talc:Lubricant:MAPE = 55:33:5:5:2 wt %. Five different BF/Talc hybrid compositions (*i.e*., 0/30, 10/20, 15/15, 20/10 and 30/0 wt % of the total shell weight) were used in the shells. Co-extruded composites with pure HDPE shell were used as the control group. The composites were manufactured with a co-extrusion system, consisting of a Leistritz Micro-27 co-rotating parallel twin-screw extruder (Leistritz Corporation, Allendale, NJ, USA) for core, a Brabender 32 mm conical twin-screw extruder (Brabender Instruments Inc., South Hackensack, NJ, USA) for shell, a die with a cross-section area of 12.7 mm × 50 mm, and a vacuum sizer [16,20]. Processing temperatures were controlled between 155 and 170 °C for the core system, and from 150 to 170 °C for shells according to different shell formulations [16]. The produced samples had a cross section dimension of 12.7 (thickness) mm × 50 (width) mm.

### 2.3. Characterization of BF/Talc/HDPE Composites

The morphology of the BF/Talc/HDPE samples was investigated using a Hitachi S4800 (Hitachi Ltd., Tokyo, Japan) scanning electron microscope (SEM). The selected samples were impact-broken after being frozen in liquid nitrogen for 10 min. The fractured sample surfaces were coated with gold before SEM observation at an acceleration voltage of 15 kV.

Flexural and tensile properties of the composites were measured based on ASTM D790-03 [21] and D638-03 [22] Standards, respectively, using an INSTRON 5582 machine (Instron Co., Grove City, PA, USA). A Tinius Olsen 92T Impact Tester (Tinius Olsen, Inc., Horsham, PA, USA) was used to test notched izod impact strengths according to the ASTM D256 [23] Standard. For each sample formulation, five replicated samples were tested. 

Dynamic mechanical analysis (DMA) of the BF/Talc/HDPE composites was done using a DMA Q800 system (TA Instruments Inc., New Castle, DE, USA) to determine composite storage modulus, loss modulus and loss factor. The tests were conducted in the linear viscoelasticity region with the maximum strain around 0.05%. A dual cantilever mode at a frequency of 1 Hz and a heating rate of 3 °C/min from −40 to 120 °C was used. The samples (60 mm × 10 mm ×3 mm) were first conditioned for 72 h at 23 °C temperature and 50% relative humidity. 

Samples with a size of 5.1 mm × 12.7 mm × 1.6 mm were used for LCTE measurement [16] using a TA Q400 ThermoMechanical Analyzer, TMA (TA Instruments Inc., New Castle, DE, USA). The length change data as a function of temperature were recorded and analyzed with the TA’s Universal Analysis software. Two heating cycles: (1) 60 to −30 °C, and (2) −30 to 60 °C were used for all samples at a fixed heating or cooling rate of 3 °C/min.

Duncan’s multiple range tests for pairwise comparison were used to test the effect of various treatments on measured data (*i.e.,* flexural, tensile and impact properties) using Statistical Analysis Software SPSS 20.0 (IBM Corp., Armonk, New York, NY, USA). Statistical ranking data at the 5% significant level was provided among the treatments for each selected property.

### 2.4. Characterization of Co-Extruded WPCs with BF/Talc/HDPE Shells

The morphology of co-extruded WPCs was studied using the Hitachi S4800 SEM (Hitachi Ltd., Tokyo, Japan). The same procedures for sample preparation and SEM analysis were followed as these used for BF/Talc/HDPE composite samples. The flexural properties of co-extruded composites were measured using a four-point bending method based on the ASTM 6272 [24] Standard using the INSTRON 5582 testing machine (Instron Co., Norwood, MA, USA). A crosshead speed of 8.7 mm/min and a span length of 250 mm were used with five replications for each sample group. Un-notched izod impact strength was measured using the Tinius Olsen Mode 1892 impact tester following the ASTM D256 [23]. Test samples (3 mm thick—five replicates of each formulation) were machined by cross-cutting the extruded profiles and tested. 

The LCTE values of each co-extruded composite type were measured using samples machined directly from extruded profiles parallel to the extrusion direction in a temperature range from 25 to −13 °C and −13 to 60°C. The specimen length was 76 ± 9 mm along the extruded direction. The samples were conditioned in an oven (or a refrigerator) at 60 °C (or −13 °C) from their initial equilibrium temperature of 25 °C for 6 h prior to size measurements with a Mitutoyo digimatic indicator (accuracy = ±0.01 mm, Mitutoyo Co., Kanagawa, Japan), and five specimens were used for each group. 

Flammability property of WPCs with BF/Talc in the shells was tested using a Standon Redcroft (Fire Testing Technology Ltd., West Sussex, UK) cone calorimeter based on the ISO 5660-1:2002 [25] Standard. All test samples were pre-conditioned and their weight and dimensions measured. For each test, the prepared sample (100 mm length × 100 mm width) was placed inside a corundum crucible. Subsequently, the crucible was mounted horizontally on the loader (which was placed on a weighing balance) and the sample was exposed to the heat radiation of 50 kw/m^2^, corresponding to a temperature of 780 °C on the upper surface of the test sample. Three replicated samples from each group were done for each group and the variability of reported data was about ±7%.

## 3. Results and Discussion

### 3.1. BF/Talc/HDPE Composite

#### 3.1.1. Basic Morphology and Bending Properties

**Figure 1:**
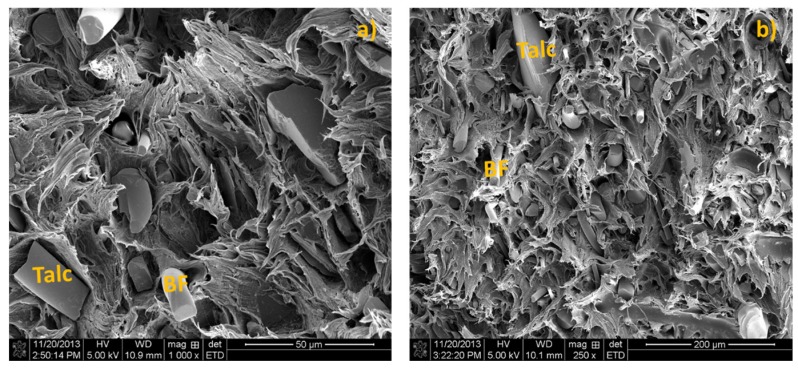
Morphology of basalt fiber (BF) and talc filled high density polyethylene (HDPE) composites. (**a**) BF/Talc = 4:36 wt % and (**b**) BF/Talc = 20:20 wt %.

Micrographs of impact fractured surfaces of the BF/Talc/HDPE composites at two different filler combination levels are shown in Figure 1. For composites with more talc loading (Figure 1a), the bulk of failure occurred due to the talc-particle pull-out from the matrix. The interfacial interactions between the talc and matrix were not strong enough to resist the pulling-out force during the fracturing process. A poor interface between talc and HDPE resulted from the existence of unmodified talc particles that did not allow much of the interaction among the two and made the particle slippage easy during the pulling-out. In general, BFs were well distributed in the matrix (Figure 1b), with a good interfacial bonding between the fiber and HDPE matrix. Most BFs were well in-bedded in the HDPE matrix as observed from sample fracture surface. Static mechanical properties of the BF/Talc/HDPE composites are summarized in Table 1. 

The data showed that the incorporation of BFs into Talc/HDPE composites improved the flexural properties, tensile modulus, and impact strength of the hybrid composites. Flexural properties of BF/HDPE composites exhibited an increasing trend as the BF content increased. The use of well dispersed and high strength BFs accounted for the improved flexural properties of the hybrid composites. In addition, the good interface interaction between BF and HDPE matrix, as observed from the SEM image, effectively helped transfer stresses from plastic matrix to the reinforcing fiber. Tensile modulus of BF/Talc/HDPE hybrid composites also increased as the BF content increased. The tensile strength of hybrid composites did not show additional increase the BF content increased. As the BF content increased, the impact strength of composites also showed an increasing trend, indicating a better interface interaction between BFs and HDPE compared to that between talc and HDPE. 

**Table 1:** Mechanical properties of the basalt fiber (BF)/Talc/ high density polyethylene (HDPE) composites.

BF/Talc (wt %)	Flexural		Tensile		Impact Strength (KJ/m^2^) ^a,b^
	Strength (MPa) ^a,b^	Modulus (GPa) ^a,b^	Strength (MPa) ^a,b^	Modulus (GPa) ^a,b^	
0/40	29.00 (0.80)A	2.35 (0.08)A	22.22 (0.56)B	3.24 (0.17)A	4.60 (0.39)A
4/36	30.38 (0.51)B	2.39 (0.08)AB	21.37 (0.55)A	3.21 (0.11)A	4.87 (0.17)AB
8/32	31.27 (0.34)C	2.48 (0.04)BC	21.33 (0.62)A	3.46 (0.07)B	5.01 (0.16)B
12/28	32.16 (0.50)D	2.49 (0.04)C	21.53 (0.51)AB	3.60 (0.18)B	5.13 (0.12)B
20/20	32.44 (0.55)EF	2.58 (0.08)D	21.79 (0.75)AB	3.88 (0.11)C	5.49 (0.25)C
40/0	33.11 (0.37)F	2.75 (0.05)E	21.31 (0.27)A	4.11 (0.09)D	6.70 (0.25)D

^a^ Values in parentheses are standard deviation based on five specimens. ^b^ Mean values with the same capital letter for each property are not significantly different at the 5% significance level.

#### 3.1.2. Surface energy Estimation

The surface energy of material is a crucial property in determining polymer wetting on the surface, which governs the interface characteristics of the two adhering surfaces [26]. The strength of the interface between the reinforcing agent and the matrix plays an important role in determining the efficiency of stress transferred from matrix to the reinforcing agent. The strength of the interface in turn governs the overall strength of the composite. A complete wetting of the matrix to the fiber or particulate surface provides an opportunity to have an intimate contact and thus adhesion between the two. Wetting is, thus, an interfacial phenomenon that is governed by the surface energy or tension of the two interacting phases, in this case matrix (HDPE) and fiber (BF) or inorganic particulate (Talc).

Surface energy of the material is defined as sum of the two main components that are based on the molecular interactions. These are based on polar (γ*^p^*) and nonpolar/disperse (γ*^d^*) interaction energy components. They are referred as Lewis acid-base (γ*^AB^*) and Liftshitz-van der Waals (γ*^LW^*) interactions refined by Van Oss *et al.* [26]. γ*^AB^* is further refined into the acid ($\gamma^{⊕}$) and base (γ^Θ^) components and calculated from their geometric mean [27,28,29]:

(1) $$\gamma^{AB}=2\sqrt{\gamma^{⊕}}\times \sqrt{\gamma^{\Theta }}$$

The total or net surface energy (γ) is the sum of the γ*^LW^* and γ*^AB^*:

(2) $$\gamma =\gamma^{LW}+\gamma^{AB}$$

The surface energy values of HDPE, BF and Talc are listed in Table 2 [28,30,31,32]. 

**Table 2:** Surface energy parameters of HDPE, BF and Talc from published literature [28,30,31,32].

Phase	γ (mN/m)	γ*^LW^* (mN/m)	$\gamma^{⊕}$ (mN/m)	γ^Θ^ (mN/m)	γ*^AB^* (mN/m)
HDPE	40.9	32	-	-	8.9
BF	140–240	61	159	11	83.64
Talc	47.7–53.3	45.5	0.02	57.01	2.14

Interfacial strength of the reinforcement and matrix is well correlated with the interfacial energy and interfacial tension [33]. A generalized expression for an estimation of the interfacial tension between the two condensed interfaces is given by Giese and Van Oss [28,34,35]:

(3) $$\gamma_{XY}={\left(\sqrt{\gamma_{X}^{LW}}-\sqrt{\gamma_{Y}^{LW}}\right)}^{2}+2\left(\sqrt{\gamma_{X}^{⊕}\gamma_{X}^{\Theta }}+\sqrt{\gamma_{Y}^{⊕}\gamma_{Y}^{\Theta }}-\sqrt{\gamma_{X}^{⊕}\gamma_{Y}^{\Theta }}-\sqrt{\gamma_{Y}^{⊕}\gamma_{X}^{\Theta }}\right)$$

where *LW* are the Liftshitz-van der Waals interactions, ⊕ and Θ are the polar interactions and *X*, *Y* are the interacting phases.

The interfacial strength can also be quantified from the thermodynamic work of adhesion (*W_A_*) between the two interacting phases. *W_A_* was related to surface tension components of the two interacting phases [36,37,38]. *W_A_* is taken as the geometric mean of the polar and the nonpolar interactions given by the following Equation:

(4) $$W_{A}=2\sqrt{\gamma_{X}^{LW}\times \gamma_{Y}^{LW}}+2\sqrt{\gamma_{X}^{AB}\times \gamma_{Y}^{AB}}$$

Table 3 provides the interfacial tension and thermodynamic work of adhesion between the HDPE-BF and HDPE-Talc interfaces calculated using the above equations. The theoretical values of the interfacial surface energy and *W_A_* of the HDPE-BF interface are higher than these of the HDPE-Talc interface. This suggests positive and better interfacial interactions of HDPE with BF than with talc. A higher interfacial energy or work of adhesion led to a stronger interface between the two phases. This was reflected in the improved impact and flexural strength of the HDPE with the BF addition and is verifiable from the SEM micrographs of the fractured specimens of the hybrid composite. High γ value of the BF provides an opportunity for the low γ HDPE matrix to completely wet its surface, which led to more intimate interactions of the two surfaces. At the higher temperature (melt temperature), γ of the polymeric materials further reduces [34], thus enhancing the wettability of HDPE. The observed stronger interfacial interaction between the HDPE and BF surface are possibly due to the contributions from high acidic surface energy ($\gamma^{⊕}$) component of BFs. For the purpose of theoretical evaluation, the surface energy values of all the materials were adapted from the different sources in literature. The marked variation in the γ of BFs is also due to its dependence on the type of processing (grinding/milling), geographical location of resource, size of particle, ratio of exposed faces (basal/lateral, lateral faces contribute to γ*^AB^* and basal to γ*^LW^*) and methods of evaluation (experimental as well as theoretical). Another approximation taken for the $\gamma_{XY}$ and *W_A_* calculations was the assumption of equal contribution of $\gamma^{⊕}$ and ($\gamma^{⊕}$ = $\gamma^{\Theta }$) to the γ of HDPE and BFs.

It should be pointed out that the above analysis is only based on theoretical calculation using published surface energy data for the studied raw materials (*i.e.,* HPDE, Talc, and BFs), and no actual experimental measurements were carried out in this study to verify the calculated data. 

**Table 3:** Calculated interfacial energy and thermodynamic work of adhesion for HDPE/Talc and HDPE-BF composites.

Interface Type	$\gamma_{XY}$ (mN/m)	*W_A_* (mN/m)
HDPE-Talc	20.2	85.0
HDPE-BF	29.9	142.9

#### 3.1.3. DMA Properties

**Figure 2:**
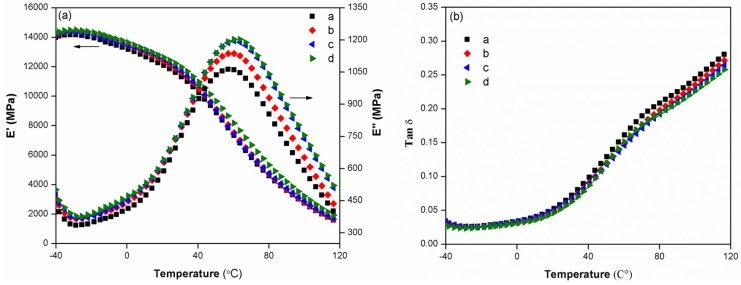
Temperature dependence of storage modulus E' and loss modulus E" (**a**) and loss tangent tanδ (**b**) with varying composition levels of BF in BF/Talc/HDPE hybrid system. a. BF/Talc (0/40), b. BF/Talc (4/32), c. BF/Talc (12/18), and d. BF/Talc (20/20).

Figure 2 shows measured storage modulus E’, loss modulus E’’ and loss tangent, tanδ, of BF/Talc/HDPE composites with varying BF/Talc contents as a function of temperature. For the BF/Talc hybrid composites with a fixed total content of BF and Talc, a slight increase trend of storage modulus with the increased BF content in the composite was observed. The storage modulus of all BF/Talc/HDPE composite decreased with an increase in temperature, converging to a narrow range at high temperatures. The incorporation of BFs imposed more mechanical limitations than Talc, thereby reduced the mobility and the deformation of the matrix with increased temperatures. For the loss modulus of composites, E’’ increased as the BF content increased and had a peak in the transition region around 50–60 °C, known as α-relaxation of HDPE [39]. The α-relaxation is considered as a complex multi-relaxation process due to the molecular motion of PE crystalline region [40]. The loss modulus at this relaxation region varied with changes of BF/Talc ratio in the composite due to varying constraints on the segmental mobility of polymer molecules introduced by different fillers. For the loss tangent of composites, the BF/Talc/HDPE composites showed a slightly decreased value of tanδ as the BF content increased, indicating that the use of BFs gave the composite more prominent elastic nature compared with talc filled composites.

#### 3.1.4. Thermal Expansion Property

**Figure 3:**
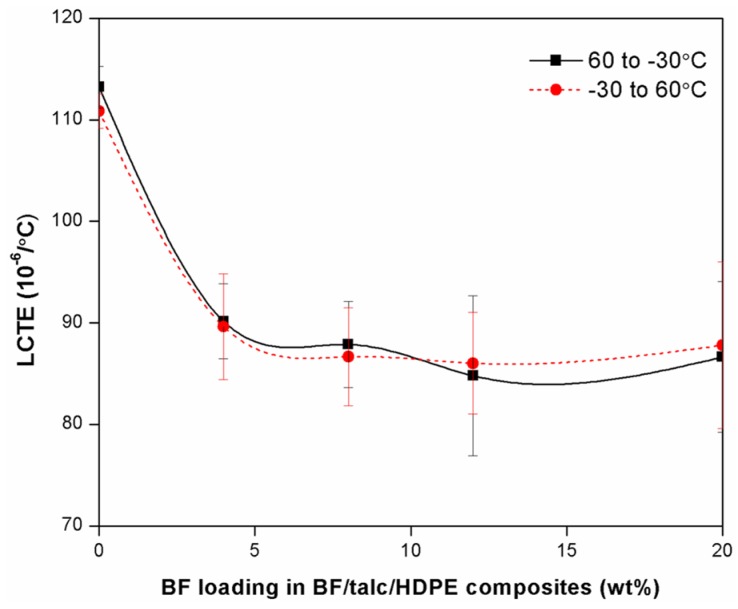
Effect of BF content on LCTE value of BF/Talc/HDPE composites.

The LCTE of fiber reinforced plastic composites is affected by the mismatch of high LCTE of the plastic matrix and low LCTE of fibers. A low LCTE value is a desirable property for composites in order to achieve dimensional stability. The measured LCTE values of BF/Talc/HDPE composites over two temperature ranges (*i.e*., 60 to −30 °C; and −30 to 60 °C) are shown in Figure 3 as a function of BF content in the BF/Talc mixture. The cooling and heating cycles led to almost identical LCTE values at each given BF content level. Generally, the LCTE values of BF/Talc/HDPE composites showed a decreasing trend as the BF loading level increased. The result was attributed to the lower LCTE of BFs and also to the fact that BFs were well-bonded to the plastic matrix, which posed a mechanical restraint on the opening or closing of the polymer chains during the heating or cooling cycles and thus helped decrease the overall LCTE of composite. A noticeable reduction (almost 20% in LCTE value) was observed when BF content increased from 0 to 4 wt %. The LCTE value of BF/Talc/HDPE composites with 20 wt % BFs were, respectively 86.6 × 10^−6^/°C and 87.8 × 10^−6^/°C from the cooling and heating cycles. However, these values are still higher than the reported LCTE values of well-made commercial WPCs filled with wood flour and other fillers. It is believed that improved interphase adhesion through surface modification of the filler can help improve the thermal expansion behaviors of filled composites with hybrid fillers [1].

### 3.2. Co-Extruded WPCs with BF/Talc/HDPE Shells

#### 3.2.1. Morphology

**Figure 4:**
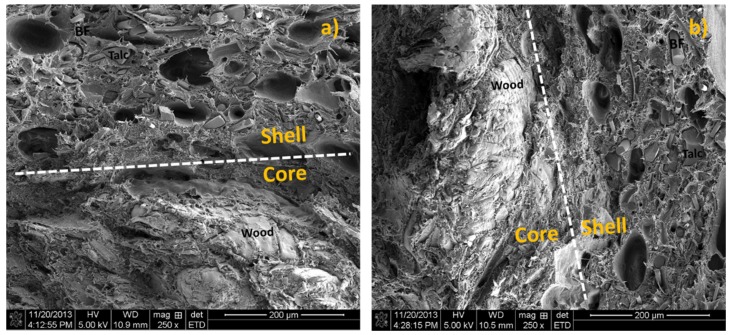
Morphology of co-extruded wood plastic composite (WPC). (**a**) BF/Talc in shell = 10:20 wt % and (**b**) BF/Talc = 20:10 wt %.

Figure 4 shows the morphology of co-extruded composites. The shell contained combined BF and talc fillers, while the core was filled mainly with wood fibers. The interface between the core and shell layers cannot be distinctively differentiated due to the use of the same matrix system (*i.e*., HDPE), indicating a good bonding between the two layers. The shell filled with more talc particles (Figure 4a) shows more Talc-particle pull-out during the fracture process compared with that of the shell with more BFs (Figure 4b).

#### 3.2.2. Mechanical Properties

**Figure 5:**
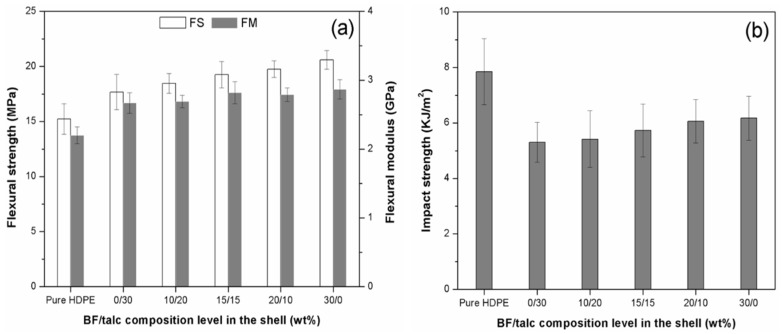
Four point bending properties (**a**) and impact strength (**b**) of co-extruded WPCs with BF/Talc filled shell. FS: flexural strength; FM: flexural modulus.

The effect of six different BF/Talc composition levels (*i.e*., 0/0, 0/30, 10/20, 15/15, 20/10 and 30/0 wt %) in the shell layer on flexural properties of co-extruded composites is shown in Figure 5a. The coextruded structure design with filled shell layers improved flexural strength and modulus of composites. Compared with the WPCs with pure HDPE shell (flexural strength = 15.23 MPa), the maximum value of flexural strength for co-extruded composites reached to 20.66 MPa when the BF loading was 30 wt %. Thus, the shell layer played a significant role in restricting the deformation of core layer and preventing the cracks from propagating to the center of the sample under loading. As the BF loading increased in the total content of hybrid fillers, the flexural modulus of the co-extruded composites also slightly increased compared with composites having pure HDPE shell.

Impact strength of the WPCs with different shell compositions is shown in Figure 5b. Compared with the composites having a pure HDPE shell, a noticeable decrease in impact strength of coextruded composites with 30 wt % overall filler content was observed. The shell layers, which contained more filler component, made the overall composites more brittle. As BF loading in the shell layer increased, the shell layer became less brittle due to increased interface interaction between BFs and matrix, resulting in the increase of impact strength for the overall composites. 

#### 3.2.3. Thermal Expansion Property

**Figure 6:**
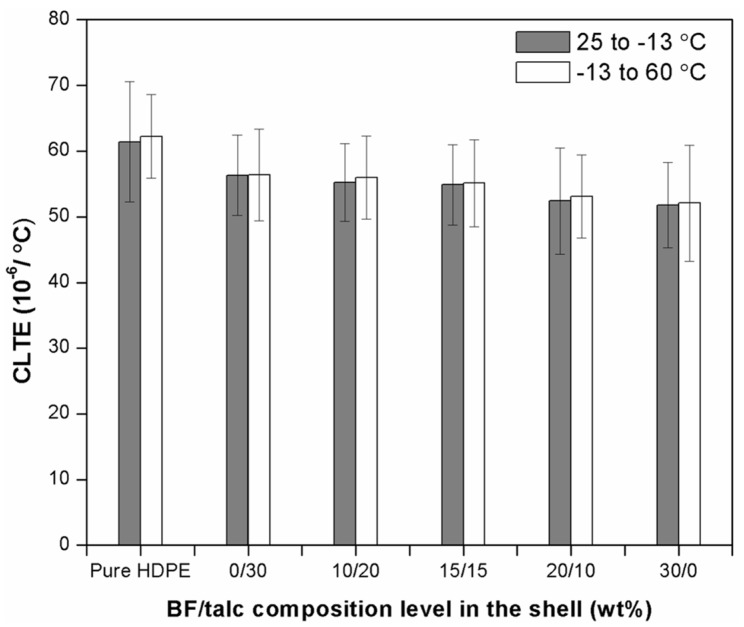
LCTE of co-extruded WPCs with different BF/Talc composition levels in the shell.

Figure 6 shows the LCTE values for the co-extruded WPCs with pure HDPE shell and hybrid BF/Talc fillers in the shells from a heating cycle (−13 to 60 °C) and a cooling cycle (25 to −13 °C). WPCs with pure HDPE shell material had the LCTE values of 61.42 × 10^−6^/°C and 62.23 × 10^−6^/°C for 25 to −13 °C and −13 to 60 °C, respectively. The LCTE values decreased to 56.32 × 10^−6^/°C and 56.38 × 10^−6^/°C for co-extruded composites with 30 wt % talc in the shell with cooling and heating cycles, respectively. As the BF content increased in the shell, the LCTE values for the co-extruded composite system showed a decreasing trend. At the 30 wt % BF level, the composite LCTE values decreased to 51.75 × 10^−6^/°C and 52.07 × 10^−6^/°C, respectively, for the two thermal treatment cycles. These values were much closer to the corresponding core-only LCTE values. Thus, incorporating BFs with good thermal stability properties in the shell layer can effectively decrease the LCTE value of the overall composite.

#### 3.2.4. Flammability Property

**Figure 7:**
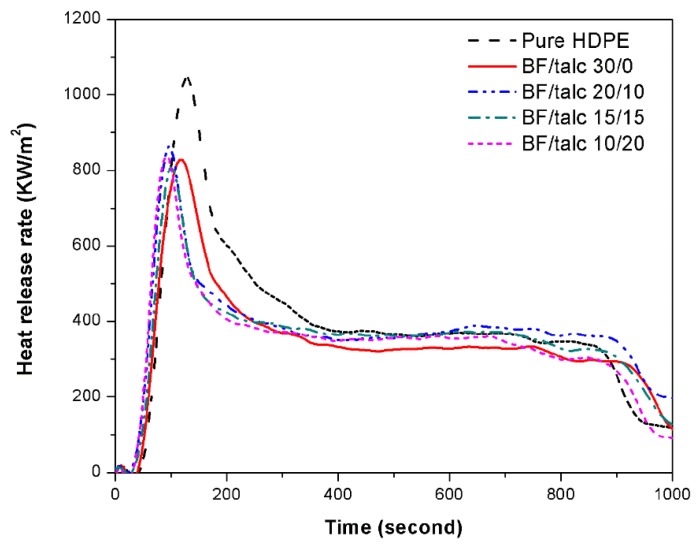
A comparison of the heat release rate (HRR) of co-extruded WPCs with different BF/Talc compositions in the shell layers.

Testing data from cone calorimetry, including peak heat release rate (PHRR), average heat release rate (AHRR), total heat released (THR), average mass loss rate (AMLR), average specific extinction area (ASEA), and average effective heat of combustion. (AEHC), are summarized in Table 4. The plots of PHRR *versus* time are shown in Figure 7. The addition of BFs in the shell layer reduced the PHRR significantly (Table 4). 

**Table 4:** Cone calorimetry data for the WPC core and co-extruded WPCs with different BF/Talc/HDPE shells.

WPC with HDPE/BF/Talc	Cone Calorimetry Data ^a^					
	PHRR KW/m^2^	AHRR KW/m^2^	THR MJ/m^2^	AMLR g/(m^2^s)	ASEA m^2^/kg	AEHC (MJ/kg)
Core ^b^	507.64	303.48	259.54	14.57	420.58	25.72
0/0 ^c^	1082.7	380.50	397.95	15.54	455.57	30.12
30/0 ^c^	869.93	340.97	349.51	13.98	395.44	28.56
20/10 ^c^	877.21	336.50	368.84	13.99	423.97	32.95
15/15 ^c^	878.36	330.89	367.74	14.07	460.56	29.44
10/20 ^c^	883.61	321.75	380.42	14.47	426.21	29.20

^a^ PHRR = peak heat release rate; AHRR: average heat release rate; THR = total heat release; AMLR = average mass loss rate; ASEA: average specific extinction area; AEHC: average effective heat of combustion. ^b^ Core only WPC. ^c^ Co-extruded WPCs with HDPE shell containing different BF/Talc compositions.

The core-only WPCs with the high filler level (*i.e*., 55 wt % WF) had the smallest heat release rate during combustion due to wood charring. For the co-extruded WPCs with a pure HDPE shell over the WPC core, its PHRR was as high as 1082.74 KW/m^2^, indicating the negative effect of the HDPE shell on the flammability property of overall composite. However, the PHRR of overall composite decreased with the use of fillers in the shell layer. At the BF loading in the shell of 30 wt %, the composite PHRR was reduced to 869.93 KW/m^2^ (19.7% reduction). In addition, the THR and AMLR values were also reduced as the addition of the BFs in the shell layer. In the composites with the combined filler system for the shell, the flame resistance was somewhat comparable among the combinations used. In general, the resistance was slightly reduced as the talc portion increased. For example, at the 10/20 BF/Talc combination, the PHRR increased to 883.61 from the 869.93 KW/m^2^ for the 30/0 BF/Talc combination. Thus, the use of BFs in the shell layer helped promote a better incomplete combustion of the co-extruded WPCs (compared with talc), leading to a better enhanced flame resistance of the composites. On the other hand, talc is more economical to use, which can help lower the overall composite costs. Thus, an optimized combined shell filler system with BFs and talc could offer a balance between cost and performance for co-extruded WPCs.

It should be pointed out that most current industrial practice in co-extruded WPCs is based on the use of specialized shell/cap materials for improved UV, water, decay, anti-scratching, and anti-skidding properties. At the same time, co-extruded WPCs with reinforced shell/cap layers (e.g., using mineral-based additives) such that the cap layer can also help modify overall strength properties of the composites have also been developed and used in practice. Thus, the information presented in this work can help better understand the performance of the latter composite systems and develop new systems with much improved properties. 

## 4. Conclusions

Mechanical, morphological and thermal properties of BF/Talc/HDPE composites and co-extruded WPCs with BF/Talc filled shells were investigated. The incorporation of BFs in Talc/HDPE matrix improved flexural properties, tensile modulus, impact strength and dynamic moduli of the hybrid composites. Strain energy estimation suggested positive and better interfacial interactions of HDPE with BFs than these with talc. As the BF content increased, the LCTE value of BF/Talc/HDPE composites showed a decreased trend. The co-extruded structure design helped improve the flexural properties and impact strength of composites due to the protective shell layer. Both composite flexural properties and impact strength increased, and the LCTE value decreased as BF content increased in the hybrid BF/talc filled shells. The composite flame resistance was improved with the use of combined fillers in the shell layer, especially with increased loading of BFs. Thus, the optimized combined shell filler system with BFs and talc could offer a balance between cost and performance for co-extruded WPCs.

## References

[1] Klyosov A.A. (2007). Wood-Plastic Composites.

[2] Wechslera A., Hiziroglu S. (2007). Some of the properties of wood-plastic composites. Build. Environ..

[3] Karrad S., Lopez Cuesta J.M., Crespy A. (1998). Influence of a fine talc on the properties of composites with high density polyethylene and polyethylene/polystyrene blends. J. Mater. Sci..

[4] Huda M.S., Mohanty A.K., Drzal L.T., Misra M., Schut E. (2005). Green composites from recycled cellulose and poly(lactic acid): Physicomechanical and morphological properties evaluation. J. Mater. Sci..

[5] Weon J.I., Sue H.J. (2006). Mechanical properties of talcand CaCO_3_-reinforced high-crystallinity polypropylene composites. J. Mater. Sci..

[6] Noel O., Clark R. Recent advances in the use of talc in wood-plastic composites. Proceeding of the 8th International Conference on Wood Fiber-Plastic Composites.

[7] Huang R., Xiong W., Xu X., Wu Q. (2012). Thermal expansion behavior of co-extruded wood plastic composites with glass-fiber reinforced shells. BioResources.

[8] Huang R., Kim B.J., Lee S.Y., Zhang Y., Wu Q. (2013). Co-extruded wood plastic composites with talc filled shells: Morphology, mechanical, and thermal expansion performance. BioResources.

[9] Czigany T., Vad J., Poloskei K. (2005). Basalt fiber as a reinforcement of polymer composites. Period. Polytech. Mech. Eng..

[10] Sim J., Park C., Moon D.Y. (2005). Characteristics of basalt fiber as a strengthening material for concrete structures. J. Compos. Part B.

[11] Wang M.C., Zhang Z.G., Li Y.B. (2008). Chemical durability and mechanical properties of alkali-proof basalt fiber and its reinforced epoxy composites. J. Reinf. Plast. Compos..

[12] Botev M., Betchev H., Bikiaris D., Panayiotou C. (1999). Mechanical properties and viscoelastic behavior of basalt fiber-reinforced polypropylene. J. Appl. Polym. Sci..

[13] Liu Q., Shaw M.T., Parnas R.S., McDonnell A.M. (2006). Investigation of basalt fiber composite mechanical properties for applications in transportation. Polym. Compos..

[14] Dalinkevich A.A., Gumargalieva K.Z., Marakhovsky S.S., Soukhanov A.V. (2009). Modern basalt fibrous materials and basalt fiber-based polymeric composites. J. Nat. Fibers.

[15] Chen J., Wang Y., Gu C., Liu J., Liu Y., Li M., Lu M. (2013). Enhancement of the mechanical properties of basalt fiber-wood-plastic composites via maleic anhydride grafted high-density polyethylene (MAPE) addition. Materials.

[16] Wu Q., Chi K., Wu Y., Lee S. (2014). Mechanical, thermal expansion, and flammability properties of co-extruded wood polymer composites with basalt fiber reinforced shells. Mater. Design.

[17] Singh S., Mohanty A.K., Misra M. (2010). Hybrid bio-composite from talc, wood fiber and bioplastic: Fabrication and characterization. Compos. Part A Appl. Sci. Manuf..

[18] Moorthy S.S., Manonmani K. (2013). Fabrication and characterization of TiO_2_ particulate filled glass fiber reinforced polymer composites. Mater. Phys. Mech..

[19] Kim B.J., Yao F., Han G., Wang Q., Wu Q. (2013). Mechanical and physical properties of core-shell structured wood plastic composites: Effect of shells with hybrid mineral and wood fillers. J. Compos. Part B.

[20] Yao F., Wu Q. (2010). Coextruded polyethylene and wood-flour composite: Effect of shell thickness, wood loading, and core quality. J. Appl. Polym. Sci..

[21] American Society for Testing and Materials (ASTM) International (2003). Standard Test Methods for Flexural Properties of Unreinforced and Reinforced Plastics and Electrical Insulating Materials.

[22] American Society for Testing and Materials (ASTM) International (2003). Standard Test Method for Tensile Properties of Plastics.

[23] American Society for Testing and Materials (ASTM) International (2010). Standard Test Methods for Determining the Izod Pendulum Impact Resistance of Plastics.

[24] American Society for Testing and Materials (ASTM) International (2010). Standard Test Method for Flexural Properties of Unreinforced and Reinforced Plastics and Electrical Insulating Materials by Four-Point Bending.

[25] International Organization for Standardization (ISO) (2002). Reaction-to-Fire Tests—Heat Release, Smoke Production and Mass Loss Rate—Part 1: Heat Release Rate (Cone Calorimeter Method).

[26] Van Oss C.J., Chaudhury M.K., Good R.J. (1988). Interfacial Lifshitz-van der Waals and polar interactions in macroscopic systems. Chem. Reviews.

[27] De Meijer M., Haemers S., Cobben W., Militz H. (2000). Surface energy determinations of wood: Comparison of methods and wood species. Langmuir.

[28] Lewin M., Mey-Marom A., Frank R. (2005). Surface free energies of polymeric materials, additives and minerals. Polym. Adv. Technol..

[29] Douillard J.M., Salles F., Henry M., Malandrini H., Clauss F. (2007). Surface energy of talc and chlorite: Comparison between electronegativity calculation and immersion results. J. Colloid Interface Sci..

[30] Van der Leeden M.C., Frens G. (2002). Surface properties of plastic materials in relation to their adhering performance. Adv. Eng. Mater..

[31] Giese R., Costanzo P., van Oss C. (1991). The surface free energies of talc and pyrophyllite. Phys. Chem. Miner..

[32] Terada K., Yonemochi E. (2004). Physicochemical properties and surface free energy of ground talc. Solid State Ionics.

[33] Tu L., Kruger D., Wagener J., Carstens P. (1997). Wettability of surface oxyfluorinated polypropylene fibres and its effect on interfacial bonding with cementitious matrix. J. Adhesion.

[34] Giese R.F., Van Oss C.J. (2002). Colloid and Surface Properties of Clays and Related Minerals.

[35] Li Z., Giese R., Van Oss C., Yvon J., Cases J. (1993). The surface thermodynamic properties of talc treated with octadecylamine. J. Colloid Interface Sci..

[36] Kaggwa G.B., Huynh L., Ralston J., Bremmell K. (2006). The influence of polymer structure and morphology on talc wettability. Langmuir.

[37] Owens D.K., Wendt R. (1969). Estimation of the surface free energy of polymers. J. Appl. Polym. Sci..

[38] Kwok D., Cheung L., Park C., Neumann A. (1998). Study on the surface tensions of polymer melts using axisymmetric drop shape analysis. Polym. Eng. Sci..

[39] Liu H., Wu Q., Han G., Yao F., Kojima Y., Suzuki S. (2008). Compatibilizing and toughening bamboo flour-filled HDPE composites: Mechanical properties and morphologies. Compos. Part A.

[40] Huang Y., Jiang S., Wu L., Hua Y. (2004). Characterization of LLDPE/nano-SiO_2_ composites by solid-state dynamic mechanical spectroscopy. Polym. Test..

